# Synthesis and characterization of linear/nonlinear optical properties of graphene oxide and reduced graphene oxide-based zinc oxide nanocomposite

**DOI:** 10.1038/s41598-023-28307-7

**Published:** 2023-01-27

**Authors:** Mohsen Ebrahimi Naghani, Mina Neghabi, Mehdi Zadsar, Hossein Abbastabar Ahangar

**Affiliations:** 1 Department of Physics, Najafabad Branch, Islamic Azad University, Najafabad, Iran; 2 Department of Chemistry, Najafabad Branch, Islamic Azad University, Najafabad, Iran

**Keywords:** Optical properties and devices, Synthesis of graphene

## Abstract

In this paper, we aimed to investigate the linear and nonlinear optical properties of GO-ZnO and RGO-ZnO nanocomposites in comparison with pure GO and reduced graphene oxide (RGO). For this purpose, GO, RGO, GO-ZnO, and RGO-ZnO were synthesized and characterized by Fourier transform infrared (FT-IR), Ultraviolet–Visible (UV–Vis) absorption, X-ray diffraction (XRD) and energy dispersive X-ray spectroscopy (EDX). XRD and EDX analysis indicated the reduction of GO as well as the successful synthesis of GO-ZnO and RGO-ZnO nanocomposites. The FT-IR spectroscopy showed that absorption bands were at 3340 cm^−1^, 1630 cm^−1^, 1730 cm^−1^ and 480 cm^−1^ related to OH, C=C, C=O, and Zn–O stretching vibrations, respectively. The direct band gaps of GO, RGO, GO-ZnO and RGO-ZnO from UV–Vis spectra were at 3.36, 3.18, 3.63 and 3.25 eV, sequentially. Moreover, the third-order nonlinear optical properties were investigated using a z-scan technique with Nd: YAG laser (532 nm, 70 mW). It can be seen that the nonlinear absorption coefficient value $(\upbeta )$ increased from 5.3 × 10^–4^ (GO) to 8.4 × 10^–3^ cm/W (RGO-ZnO). In addition, nonlinear refractive index (n_2_) of the GO, RGO, GO-ZnO, and RGO-ZnO was obtained as 10.9 × 10^–10^, 14.3 × 10^–10^, 22.9 × 10^–10^, and 31.9 × 10^–10^ cm^2^/W respectively.

## Introduction

After the discovery of graphene by Geim and Noveselev in 2004, tremendous research was carried out on the field of this thinnest and flattest material that could ever be in the universe^1–4^. Graphene has offered a unique two-dimensional (2D) sp^2^-hybridized structure and great properties such as high mechanical flexibility, superior electrical and thermal conductivities, large specific surface area, and high chemical stability^5–11^. Because of these characteristics, graphene has various applications including in supercapacitors^12,13^, photovoltaics^14–16^, fuel cells^17–19^, sensors^20,21^ and nanofluids^22,23^. Moreover, 2D crystal structure of Graphene can make it more popular in order to load diverse materials or form various composites that enhance the beneficial features of both graphene and the added components^24^. For instance, it is observed that graphene/metal oxide composites have shown higher performance for energy storage^25–28^ and electrochemical detection^29–32^ in comparison with individual graphene or added components. Furthermore, research on functionalized graphene has shown that graphene composites exhibit remarkable nonlinear optical (NLO) responses^33^. In this respect, inorganic metal oxides can be good candidates for combining with graphene. Recently, they have attracted significant attention because of their wide utilization in catalysis, water purification, hydrogen production, lithium-ion batteries, and transparent electronics^34–39^. For example, Zinc oxide (ZnO) is an inorganic metal oxide with a wide band gap of 3.37 eV^40,41^ and a large exciton binding energy at room temperature (60 meV) has diverse potential applications such as light emitting diodes^42^, solar cells^43–46^, sensors^47–49^, photodetectors^50^, and nanogenerators^1,5^. Consequently, according to the super individual properties of graphene and ZnO, combining graphene with ZnO nanoparticles can enhance performances^1^. Obviously, proper solubility and processability are considered the first requirements for many applications of graphene-based materials^33^. The poor solubility of graphene limited its application in either organic solvent or inorganic solvent^24^. One of the possible methods to improve the solubility is the oxidation of graphene and modification of GO with some soluble materials. Since GO has large quantities of oxygen-containing groups such as carboxyl, carbonyl and hydroxyl/epoxy, it can easily provide various types of decoration ways with organic and inorganic materials by covalent/no covalent functionalization^51^. Graphene oxide (GO) can be synthesized by various methods such as Staudenmaier^52^, Hofmann^53^, Jaleh^54^ and Marcano^55^. Among them, the Hummers’ method is widely used for the production of GO today^56^.

As assumed, combination of graphite with metal oxides can enhance the optoelectronic properties. For this purpose, in this work we study the linear and nonlinear optical properties of GO-ZnO and RGO-ZnO hybrid. Firstly, GO was synthesized directly from graphite by a modified Hummers’ method. Then, the nanocomposite of RGO-ZnO was fabricated using the hydrothermal method. The structural properties of samples were investigated using powder X-ray diffraction (XRD), Energy-dispersive x-ray (EDX) and Fourier-transform infrared (FT-IR) spectra. Optical properties of samples were investigated by UV–Visible spectroscopy and Z-scan analysis.

The Z-scan technique studied the NLO properties of GO, RGO, GO-ZnO and RGO-ZnO. The results show that GO-ZnO and RGO-ZnO hybrids exhibit enhanced NLO properties compared to GO and RGO. Hence, GO-ZnO and RGO-ZnO nanocomposites can be good candidates for optical communication and optical storage^33^. It is worth saying that in this study a noble approach was developed to compare GO, RGO, GO-ZnO, and RGO-ZnO characteristics usefully which has not reported in many works before. In this efficient pathway all the properties of GO, RGO, GO-ZnO, and RGO-ZnO such as $\upbeta$ and n_2_ values are provided in one table (Table 1).

**Table 1:** The calculated nonlinear refractive index and nonlinear absorption coefficient using the Z-scan method.

Sample	I_0_ (w/cm)	α (1/cm)	ΔT_(P–V)_	β (cm/w)	n_2_ (cm^2^/w)
GO	0.05 × 10^5^	0.7	0.44	5.3 × 10^–4^	10.9 × 10^–10^
RGO	0.05 × 10^5^	0.3	0.58	8.3 × 10^–4^	14.3 × 10^–10^
GO-ZNO	0.05 × 10^5^	1.3	0.97	3.6 × 10^–3^	22.9 × 10^–10^
RGO-ZNO	0.05 × 10^5^	1.9	1.25	8.4 × 10^–3^	31.9 × 10^–10^

## Experimental

### Materials

Spectroscopically pure (SP) graphite, sulfuric acid (H_2_SO_4_), hydrogen peroxide (H_2_O_2_), Potassium hydroxide (KOH), hydrazine hydrate (N_2_H_4_), Zinc acetate dehydrate Zn (CH_3_COO)·7H_2_O potassium permanganate (KMnO_4_) and Sodium hydroxide (NaOH) were purchased from Merck chemical company and used without any purification.

### Synthesis of graphene oxide (GO)

GO was prepared using the Hummers’ Method and by oxidation of graphite. First, 0.2 g graphite was mixed with concentrated H_2_SO_4_ (50 ml) and then stirred for 12 h. Subsequently, the mixture was cooled in an ice bath under vigorous stirring, and at the same time, KMnO_4_ (2.5 g) was added slowly to the suspension and stirred for 2.5 h. The solution was diluted with distilled water (50 ml) and the stirring process was continued for 1 h. The mixture was further allowed to cool down to room temperature and finally treated with 50 ml distilled water followed by 100 ml H_2_O_2_ 30%. Then, it was purified by centrifuging and washing with excess water until the pH reached 7 to obtain GO^56^.

### Synthesis of reduced graphene oxide (RGO)

Graphite oxide was dispersed in deionized water by sonication for 2 h. Then, 0.6 g KOH (purity 99.5%) and 4 ml hydrazine hydrate (concentration 80%) were added to the suspension and the temperature increased up to 100 °C and refluxed for 24 h. In the end, the obtained RGO was washed and dried^52^.

### Preparation of GO-ZnO and RGO-ZnO nanocomposite

For the preparation of GO-ZnO nanocomposite with the mass ratio of 10% GO, 2.7 g of Zn (CH_3_COO)·7H_2_O was added to 30 ml deionized water and then, 0.05 g GO was added under low-speed stirring. After that, NaOH 3 M was added to the above solution slowly until pH reached 12 and the resulting mixture was ultrasonicated for 30 min. The final mixture was transferred to a Teflon-lined autoclave for hydrothermal synthesis at 160 °C in an oven for 20 h. The precipitate (GO-ZnO) was washed three times with deionized water and acetone and dried at 80 °C. In order to obtain RGO-ZnO, the mentioned stages were repeated with 0.05 g of RGO.

## Results and discussion

### Structural and elemental properties

**Figure 1:**
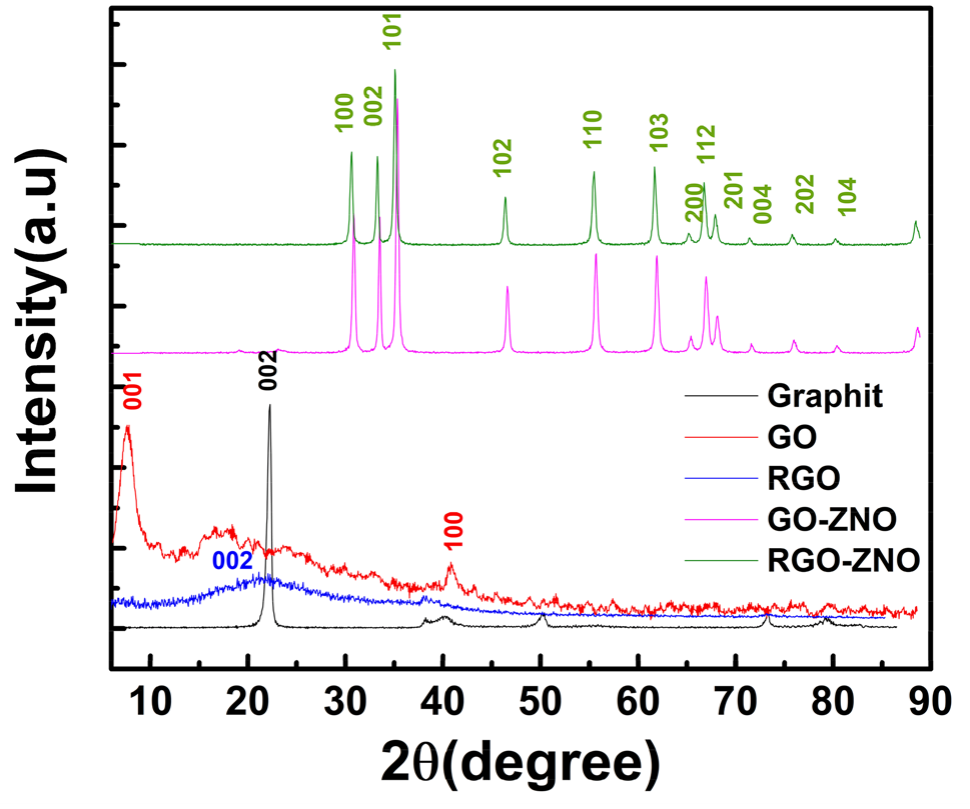
XRD patterns of Graphite, GO, RGO, GO-ZnO, and RGO-ZnO nanocomposite.

**Figure 2:**
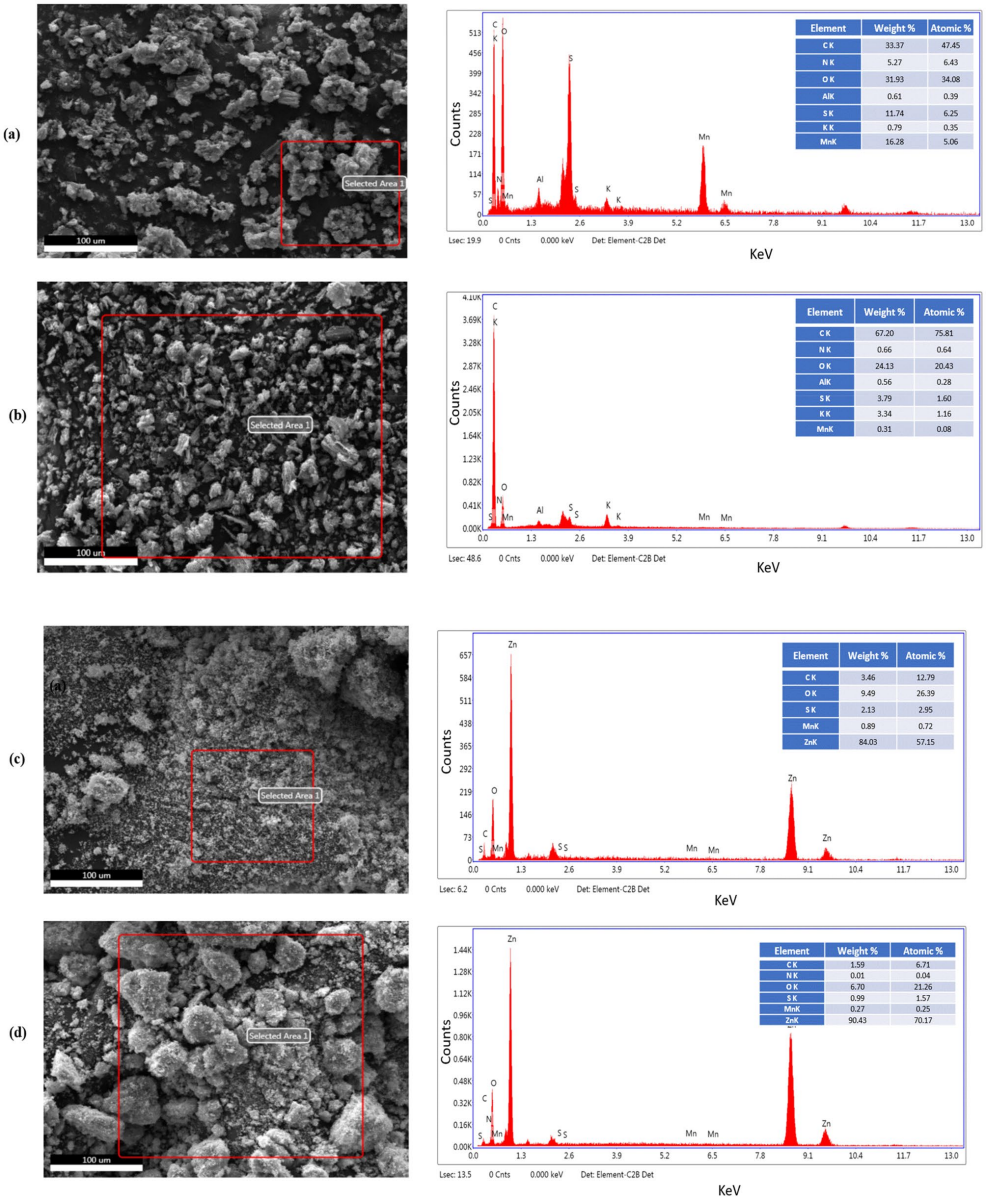
Scanning electron microscopy with energy-dispersive X-ray spectroscopy (SEM–EDX) analysis (**a**) GO, (**b**) RGO, (**c**) GO-ZnO, and (**d**) RGO-ZnO.

Crystal structure quality and orientation of Graphite, GO, RGO, GO-ZnO, and RGO-ZnO nanocomposite are shown in Fig. 1. The XRD pattern of GO shows the sharp diffraction peak at 9° corresponds to the basal spacing of GO (8.97 Å) and due to the intercalation of oxygen-containing groups, it is higher than Graphite (4 Å)^5^. In graphite pattern, the sharp peak at 2θ = 26.4 and wide peak at 24.42 in RGO pattern are attributed to the (002) plane of a carbon atom. This wide diffraction peak in RGO is related to the poor ordering of the sheets along the plane direction and many defects in the carbon lattice. The GO-ZnO and RGO-ZnO nanocomposite patterns indicate highly crystalline peaks at 2θ = 31.9°, 34.7°, 36.5°, 47.7°, 56.8°, 63.0°, 66.6°, 67.9°, 69.2°, 72.7°. They can be attributed to the high crystallinity of ZnO during the hydrothermal method led to the disappearing characteristic peak of GO and RGO. Moreover, the results of Energy-dispersive X-ray (EDX) spectroscopy are provided in in Fig. 2 which show the present of Zn, C, S, Mn, K, and O atoms in our samples. Also, the elemental distribution and EDS spectrum indicates a clear change of Oxygen groups ratio from 34.08 (at%) in GO to 20.43 (at%) in RGO samples^5,30^.

### FT-IR

**Figure 3:**
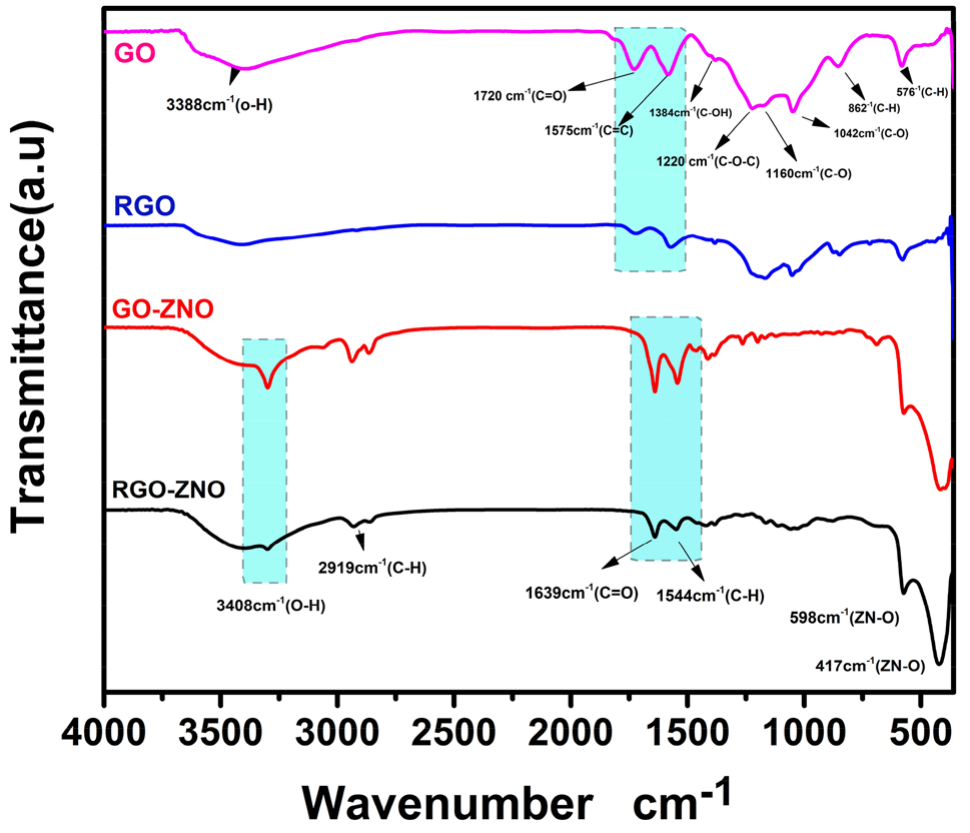
FTIR spectra of GO, RGO, GO-ZnO, and RGO-ZnO.

The results of FTIR spectroscopy of GO, RGO, GO-ZnO, and RGO-ZnO are depicted in Fig. 3. The pattern of GO shows an O–H group stretching vibration band at 3340 cm^−1^. The sp^2^ structure of C=C and carbonyl functional groups (C=O stretching) are observed at 1630 cm^−1^ and 1730 cm^−1^ respectively^24^. The absorption peak at 1220 cm^−1^ and 1044 cm^-1^ are related to the C–O stretching vibration band. It must be mentioned, the vibrations bands of O–H, C=O, and C–O have been severely reduced, attenuated and slightly shifted to a lower wavenumber due to deoxygenation in RGO. Finally, the absorption peaks at 480 cm^−1^ in GO-ZnO and RGO-ZnO patterns can be attributed to ZnO stretching vibration.

### UV–Vis absorption spectra

**Figure 4:**
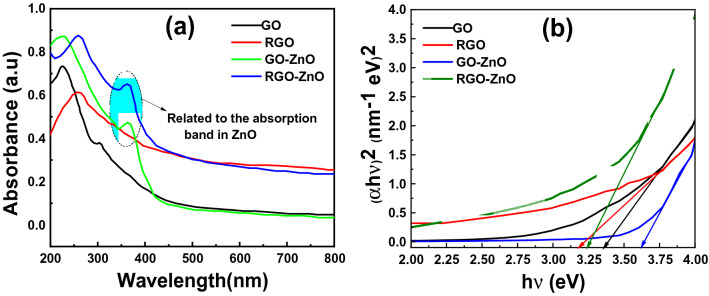
(**a**) Absorbance spectra of GO, RGO, GO-ZnO, and RGO-ZnO nanocomposite and (**b**) band gap determination according to absorption measurements of GO, RGO, GO-ZnO, and RGO-ZnO.

As shown in Fig. 4a, the UV–Vis spectra show that GO exhibits two absorption peaks: one at about 230 nm, presumably due to the π → π* transition of the C–C bonds, and another shoulder at about 300 nm corresponds to the n → π* transition of the C = O bonds^57–60^. Whereas the peak of the π → π* transition shifts to 260 nm for RGO, suggesting that some groups on the GO surface are removed and the conjugated structure is restored, reflecting increased π-electron concentration and structural ordering, which is consistent with the restoration of sp^2^ carbon and possible rearrangement of atoms^61,62^. Two absorption peaks were observed in the spectrum of GO-ZnO at 230 nm and 366 nm related to the GO absorption peak and the main absorption peak of ZnO respectively^63^. RGO–ZnO hybrids exhibit band edge absorption at 361 nm, almost 9 nm less blue-shifted than that of the band gap absorption of bulk ZnO at 370 nm, which might be explained by the quantum confinement effect of the smaller feature size of ZnO. Also, it can be seen a slight blue shift in absorption peak of RGO-ZnO from 260 to 258 nm comparing pure RGO.

The optical absorption coefficient ($\mathrm{\alpha }$) can be calculated using Eq. (1),

1 $$\mathrm{\alpha h\nu }=\mathrm{D}{\left(\mathrm{h\nu }-{\mathrm{E}}_{\mathrm{g}}\right)}^{\mathrm{n}},$$

where D is constant, $\mathrm{h\nu }$ is the incident photon energy and ${\mathrm{E}}_{\mathrm{g}}$ is the optical band gap and n illustrates type of optical transition. In the present case, n = 1/2 is considered, which is attributed to direct bandgap of prepared material; hence, the plot of ${\left(\mathrm{\alpha h\nu }\right)}^{2}$ versus $\mathrm{h\nu }$ is depicted in Fig. 4b. Finally, the bandgap of prepared materials was calculated by extrapolation used on X-axis.

The band gaps of GO and RGO are ~ 3.36 eV and 3.18 respectively because on reduction, some of the oxygen groups are removed and bandgap can therefore be adjusted further by managing the oxygen present in RGO. The Energy bandgap for pure ZnO found 3.37 eV^64^ and with GO, it increased around 3.63 eV. It is well-known that the excitation energy of ZnO nanoparticles will increase with the decrease of grain diameter according to the Kubo theory^65^; therefore, the blue shift of ZnO bandgap in GO-ZnO structure can be attributed to the reduction of ZnO nanoparticles size. The band gap energy of RGO-ZnO reduced to 3.25 eV due to the increase in the surface charge between ZnO and RGO led to the optical band gap shifting to a higher wavelength.

### Nonlinear optical measurement

As the NLO properties are of great importance for high performance all-optical photonic devices, in this part, the third-order optical nonlinearities of the GO, RGO, GO-ZnO and RGO-ZnO samples have been investigated using the Z-scan techniques. Therefore, the second harmonic of a Q-switched Nd: YAG laser (532 nm, 4 ns) was used as the laser source. The concentrations of the sample solutions GO, RGO, GO-ZnO and RGO-ZnO are 0.2 mg/ml, which were placed in 1 mm quartz cells. After entering the sample, the laser beam was divided by a beam splitter. The measurements were carried out employing open-aperture and closed-aperture configurations. The reflected beam was used as an open-aperture signal and the transmitted one passed through a small hole (s = 0.3) as a close-aperture signal.

### Nonlinear optical properties

As Graphene has excellent nonlinear optical properties and optical limiting performance, it was of significant interest to assess the NLO properties of metal oxides combined with graphene. Graphene has a unique atomic and electronic 2D sheet structure including sp^2^ hybridized carbon atoms. On the other hand, GO is a mainly 2D network which contains a large number of sp^3^ hybridized carbon atoms along with some sp^2^ domains and its concentration can be increased by chemical reduction. It is reported that highly reduced GO with a larger percentage of sp^2^ carbon domains exhibit good SA, while partially reduced GO exhibit good RSA characteristics^66^.

Due to the existence of prior graphitic nanoislands which are sp^2^-hybridized carbon clusters, the GO possess some properties of graphene. For example, ultrafast carrier dynamics and Pauli blocking, cause fast SA in ultra-broad spectra region. As a result, after being excited by a 532 nm laser, the SA which originates from Pauli blocking overcomes the NLO absorption at low pump intensities. According to the small amount of the sp^2^ configurations in GO, the contribution of excited state absorption (ESA) originating from small localized sp^2^ configurations to the nonlinear absorptive valley should be minor compared to the TPA^67^.

The nonlinear absorption (NLA) of GO is mostly derived from two-photon absorption (TPA), originating from the sp^3^ domains; it dominates the NLA at high pump intensities which is because of the high energy gap of sp^3^ bonded carbon (2.7–3.1 eV)^68^.

**Figure 5:**
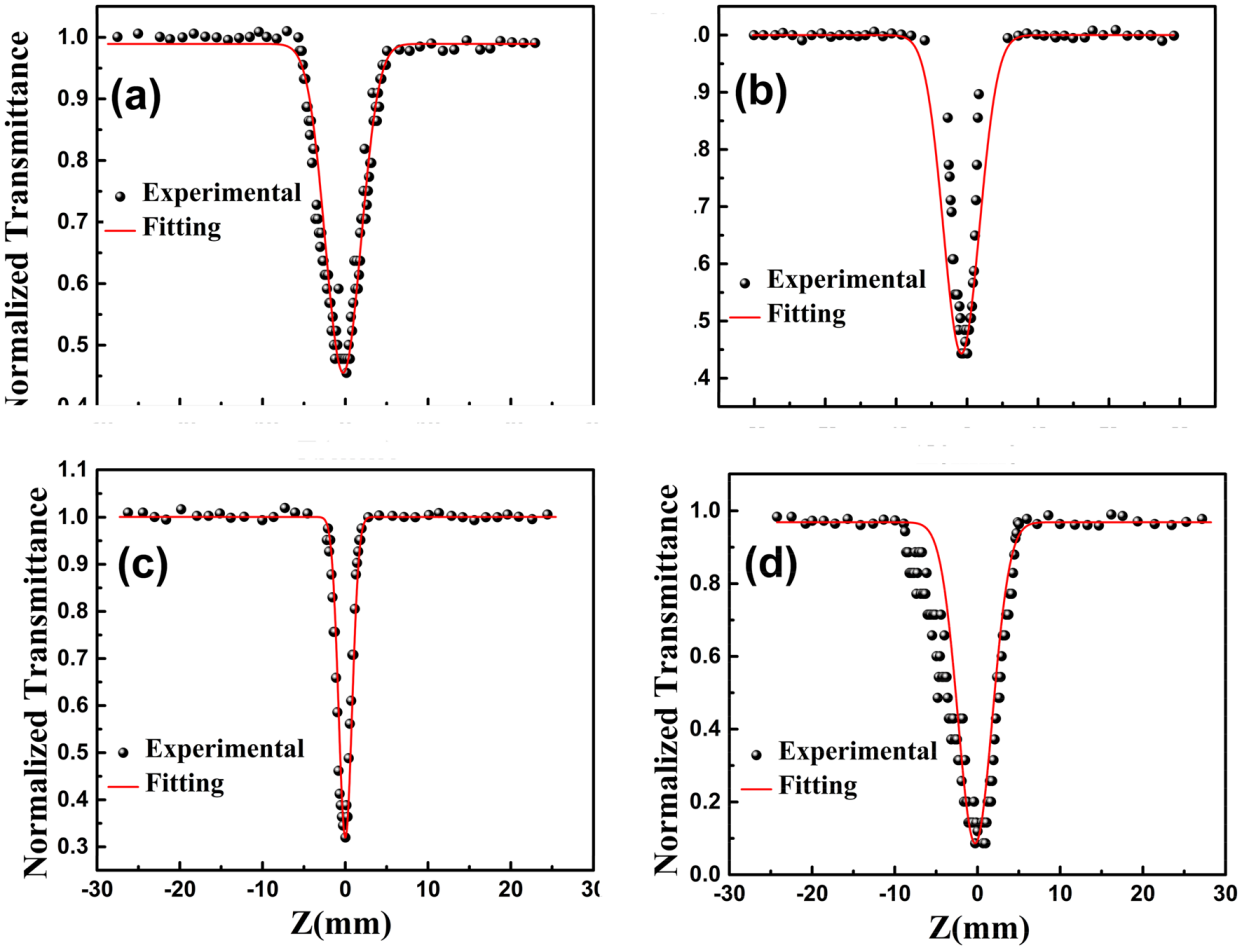
Open aperture measurements (at 532 nm) of (**a**) GO, (**b**) RGO, (**c**) GO-ZnO, and (**d**) RGO-ZnO at excitation intensity of 532 nm. Symbols represent experimental data and solid lines represent theoretical fit.

Figure 5 shows open-aperture Z-scan results of GO, RGO, GO-ZnO and RGO-ZnO. It is known that the valley depth of the Open aperture Z-scan curve reflects the optical limiting properties of the material. If the valley was deeper, its optical limiting performance would be better^68^.

As the figure shows, the normalized transmittance curve of GO, RGO, GO-ZnO, and RGO-ZnO samples include valleys, which means that they exhibit reverse saturable absorption (RSA). Obviously, the depths of the valleys in the GO and RGO curves are slightly different which demonstrates that reduction process in RGO can improve NLO properties of GO.

In addition, the nonlinear absorptive valley of RGO–ZnO is obviously deeper and broader than that of GO–ZnO, which suggests an increase in NLO properties^68^. It can be mostly attributed to the thermal reduction of the GO moiety to RGO. Following the reduction, the small localized sp^2^ configurations may increase greatly in number but cannot interconnect to form new sp^2^ carbon clusters in RGO moiety^67^. The nonlinear absorption coefficient β of GO, RGO, GO-ZnO, and RGO-ZnO was investigated. The curves of materials show different trends for nonlinear absorption coefficient β, which should be owing to their complicated NLO response mechanisms. After covalent functionalization with ZnO, the GO–ZnO hybrid exhibits a much higher value of β than that of GO. Moreover, the RGO–ZnO hybrid shows the significantly larger value of β regarding that of the GO–ZnO hybrid and yields the highest nonlinear absorption coefficient β of 31.9 × 10^−10^ cm/w, which can be related to the effective reduction of GO moiety to RGO. The larger value of β observed for RGO–ZnO suggests that it should have competitively better optical limiting performance^67^.

To evaluate the nonlinear optical properties of GO, RGO, GO-ZnO and RGO-ZnO quantitatively, we fit the experimental data with the following equations:

2 $$\mathrm{T}\left(\mathrm{z}\right)=\sum_{\mathrm{m}=0}^{\infty }\frac{{\left({-\mathrm{q}}_{0}\left(\mathrm{z}\right)\right)}^{\mathrm{m}}}{{\left(\mathrm{m}+1\right)}^\frac{3}{2}},$$

3 $${\mathrm{q}}_{0}\left(\mathrm{z}\right)=\frac{\upbeta {\mathrm{I}}_{0}{\mathrm{L}}_{\mathrm{eff}}}{\left(1+\frac{{\mathrm{z}}^{2}}{{\mathrm{z}}_{0}^{2}}\right)} ,$$

where T is the open aperture normalized transmittance, ${\mathrm{z}}_{0}=\frac{\uppi {\mathrm{W}}_{0}^{2}}{\uplambda }$ is the Rayleigh range, z is the sample position, W_0_ is the beam waist at the focal point (Z = 0), $\uplambda$ is the laser wavelength, I_0_ is the peak intensity, and ${\mathrm{L}}_{\mathrm{eff}}=\frac{1-\mathrm{exp}(-\mathrm{\alpha L})}{\mathrm{\alpha }}$ is the effective thickness of the sample where α is the linear absorption coefficient of the sample and is calculated from the UV spectrum^68^.

In the situation of third order nonlinearity, the refraction index of material can be expressed in terms of light intensity:

4 $$ {\text{n }}\left( {\text{I}} \right) \, = {\text{ n}}_{0} + {\text{ n}}_{{2}} {\text{I}}, $$

where n_0_ is the linear refractive index and I is the intensity of the incident laser light. The z-scan measurement with an aperture (close aperture) was performed for the investigation of nonlinear refraction of GO, RGO, GO-ZnO, and RGO-ZnO nanocomposite.

Typical peak–valley (valley–peak) transmittance curve is achieved when the nonlinear refractive index of the medium is negative (positive). For determining the nonlinear refractive index, one can monitor the transmittance change through a small circular aperture which is placed at the far-field position.

In order to obtain the relation between the normalized transmittance T(z) and z position, the samples move along the axis of the incident beam (z-direction) concerning the focal point. The variation of this quantity as a function of ΔΦ_0_ is given by:

5 $$ \Delta {\text{T}}_{{{\text{P}} - {\text{V}}}} = \, 0.{4}0{6}\left( {{1} - {\text{ S}}} \right)^{0.25} \Delta \Phi_{0} , $$

where S is the linear transmittance of the far-field aperture. |ΔΦ_0_| relates to n_2_ through the following expression:

6 $$ \Delta \Phi_{0} = {\text{ k}} L_{eff} {\text{n}}_{{2}} {\text{I}}_{0} , $$

where I_0_ is the intensity of the laser beam at focus z = 0, ${\mathrm{L}}_{\mathrm{eff}}=\frac{1-\mathrm{exp}(-\mathrm{\alpha L})}{\mathrm{\alpha }}$ is the effective thickness of the sample, α is the linear absorption coefficient and L is the thickness of the sample. The nonlinear refractive index n_2_ (cm^2^/w) can be obtained from Eqs. (5) and (6)^69^.

**Figure 6:**
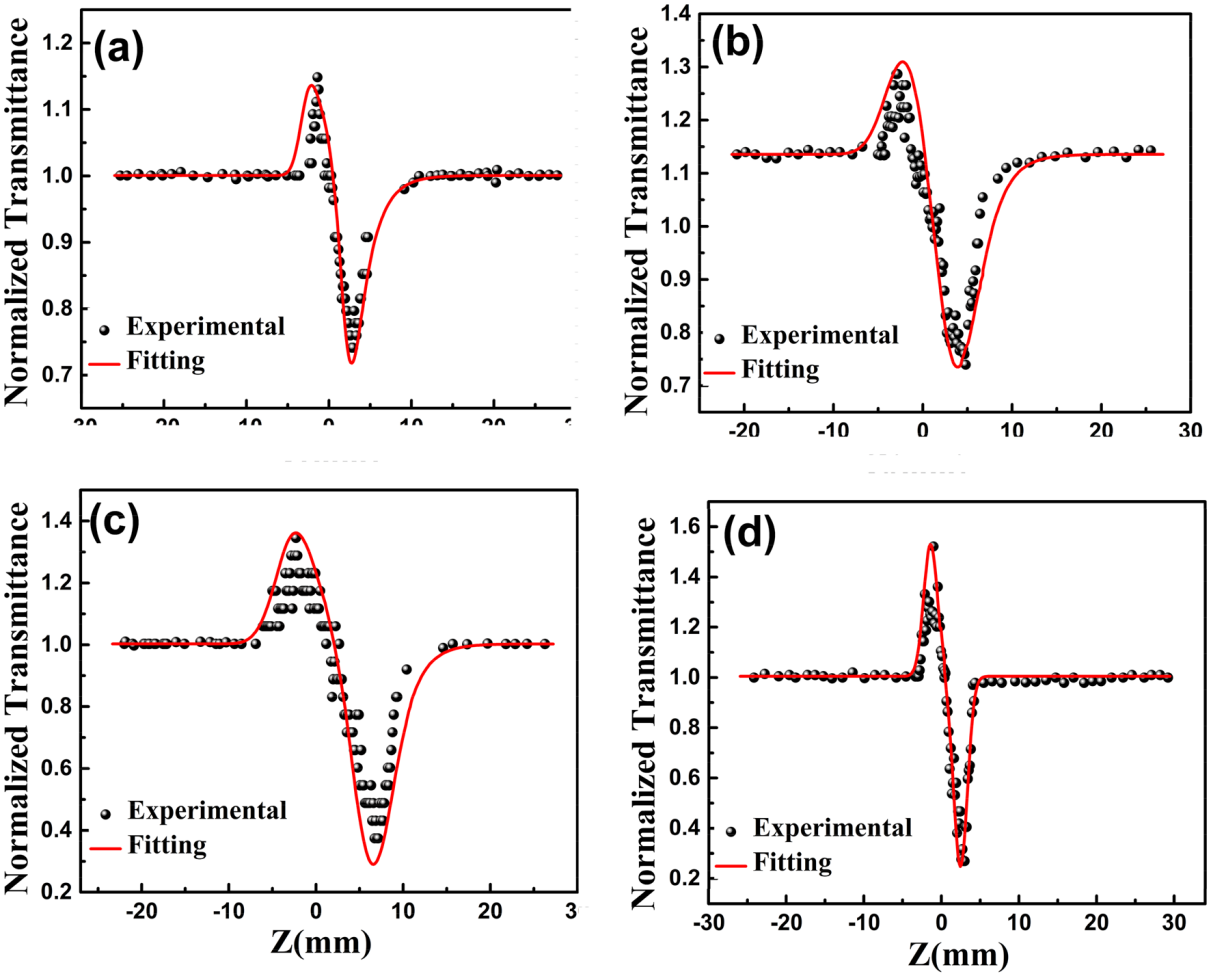
Closed aperture measurements (at 532 nm) of (**a**) GO, (**b**) RGO, (**c**) GO-ZnO, (**d**) RGO-ZnO. Symbols represent experimental data and solid lines represent theoretical fit.

The close-aperture Z-scan results of GO, RGO, GO-ZnO, and RGO-ZnO are provided in Fig. 6. In this figure, symbols show experimental transmission data, while solid lines are taken by fitting the experimental data to the non-linear transmittance. Moreover, the nonlinear refractive index (n_2_) is taken to be a fitting parameter.

Closed aperture experimental data of GO, RGO, GO-ZnO, and RGO-ZnO for excitation intensity of 532 nm are found to fit well for typical values of n_2_. It is clearly evident from Fig. 6 that the samples exhibit prefocal peak and postfocal valley characteristics, which is a direct indication of negative n_2_ (positive lens) and it suggests that RGO-ZnO can also be used as self-focusing materials around 532 nm. The difference between normalized peak and valley transmittance ΔT_P-V_ (denoting T_P_−T_V_) can be directly measured by z-scan technique^70^. The calculated values related to ΔT_P-V_ and n_2_ are provided in Table 1.

## Conclusions

We have reported the synthesis, structure and nonlinear optical properties of GO, RGO, GO-ZnO, and RGO–ZnO nanocomposite. The results of XRD, EDX, FT-IR, and UV–Vis confirm the successful fabrication of RGO–ZnO. The samples were separately characterized and tested for nonlinear optical properties. The results of open-aperture Z-scan testing on GO, RGO, GO-ZnO, and RGO-ZnO showed the significantly enhancing nonlinear absorption coefficient β values of RGO-ZnO (8.4 × 10^−3^ cm/w) compared to pure GO and GO-ZnO. It can be attributed to the combination of different NLO mechanisms in RGO–ZnO including the SA from the sp^2^ clusters in the RGO moiety and the RSA originating from the ZnO moiety. Furthermore, the Z-scan curve of RGO-ZnO displays a deeper RSA valley. the close-aperture Z-scan results of GO, RGO, GO-ZnO, and RGO-ZnO suggest a notable increase in nonlinear refractive index n_2_ of RGO-ZnO sample in comparison with GO.

Considering the easy-to-prepare and low-cost RGO-ZnO nanocomposite and its excellent NLO properties, this work may provide some insight into the design of other novel graphene-based materials for optoelectronic devices such as optical limiting, optical switches, and optical sensors.

## Data Availability

The datasets used and/or analyzed during the current study available from the corresponding author on reasonable request.

## References

[1] Kavitha T, Gopalan AI, Lee K-P, Park S-Y (2012). Glucose sensing, photocatalytic and antibacterial properties of graphene–ZnO nanoparticle hybrids. Carbon.

[2] Gupta V, Saleh TA (2011). Syntheses of carbon nanotube-metal oxides composites; adsorption and photo-degradation. Carbon Nanotubes Res. Appl..

[3] Peng C, Xiong Y, Liu Z, Zhang F, Ou E, Qian J (2013). Bulk functionalization of graphene using diazonium compounds and amide reaction. Appl. Surf. Sci..

[4] Miyaji H, Kanemoto Y, Hamamoto A, Shitomi K, Nishida E, Kato A (2022). Sustained antibacterial coating with graphene oxide ultrathin film combined with cationic surface-active agents in a wet environment. Sci. Rep..

[5] Li C, Cheng Z, Gao J, Han Q, Ye M, Zhang J (2016). Oxidation degree of graphene reflected by morphology-tailored zno growth. Carbon.

[6] Giannakopoulou T, Todorova N, Erotokritaki A, Plakantonaki N, Tsetsekou A, Trapalis C (2020). Electrochemically deposited graphene oxide thin film supercapacitors: Comparing liquid and solid electrolytes. Appl. Surf. Sci..

[7] Jain R, Sinha A (2016). Graphene-zinc oxide nanorods nanocomposite based sensor for voltammetric quantification of tizanidine in solubilized system. Appl. Surf. Sci..

[8] Wördenweber H, Karthäuser S, Grundmann A, Wang Z, Aussen S, Kalisch H (2022). Atomically resolved electronic properties in single layer graphene on α-Al2O3 (0001) by chemical vapor deposition. Sci. Rep..

[9] Hameed N, Dumée LF, Allioux F-M, Reghat M, Church JS, Naebe M (2018). Graphene based room temperature flexible nanocomposites from permanently cross-linked networks. Sci. Rep..

[10] Li Y, Zhu G, Zhou K, Meng P, Wang G (2021). Evaluation of graphene/crosslinked polyethylene for potential high voltage direct current cable insulation applications. Sci. Rep..

[11] Sodeinde K, Olusanya S, Lawal O, Sriariyanun M, Adediran A (2022). Enhanced adsorptional-photocatalytic degradation of chloramphenicol by reduced graphene oxide-zinc oxide nanocomposite. Sci. Rep..

[12] Rout CS, Govindaraj A (2008). Graphene-based electrochemical supercapacitors. J. Chem. Sci..

[13] Kim B-M, Kim H-Y, Hong S-W, Choi WH, Ju Y-W, Shin J (2022). Structurally distorted perovskite La0. 8Sr0.2Mn0.5Co0.5O3-δ by graphene nanoplatelet and their composite for supercapacitors with enhanced stability. Sci. Rep..

[14] Choi K-H, Nam H-J, Jeong J-A, Cho S-W, Kim H-K, Kang J-W (2008). Highly flexible and transparent In Zn Sn Ox/Ag/In Zn Sn Ox multilayer electrode for flexible organic light emitting diodes. Appl. Phys. Lett..

[15] Raad SH, Atlasbaf Z (2021). Solar cell design using graphene-based hollow nano-pillars. Sci. Rep..

[16] Chang J-K, Huang Y-Y, Lin D-L, Tau J-I, Chen T-H, Chen M-H (2020). Solution-processed, semitransparent organic photovoltaics integrated with solution-doped graphene electrodes. Sci. Rep..

[17] Williams G (2008). TiO2-graphene nanocomposites. UV-assisted photocatalytic reduction of graphene oxide. ACS Nano.

[18] Seger B, Kamat PV (2009). Electrocatalytically active graphene-platinum nanocomposites. Role of 2-D carbon support in PEM fuel cells. J. Phys. Chem. C.

[19] Srikanth S, Dudala S, Jayapiriya U, Mohan JM, Raut S, Dubey SK (2021). Droplet-based lab-on-chip platform integrated with laser ablated graphene heaters to synthesize gold nanoparticles for electrochemical sensing and fuel cell applications. Sci. Rep..

[20] Mashhadzadeh AH, Ahangari MG, Dadrasi A, Fathalian M (2019). Theoretical studies on the mechanical and electronic properties of 2D and 3D structures of beryllium-oxide graphene and graphene nanobud. Appl. Surf. Sci..

[21] Chang Y-S, Chen F-K, Tsai D-C, Kuo B-H, Shieu F-S (2021). N-doped reduced graphene oxide for room-temperature NO gas sensors. Sci. Rep..

[22] Baby TT, Ramaprabhu S (2010). Investigation of thermal and electrical conductivity of graphene based nanofluids. J. Appl. Phys..

[23] Tao H, Alawi OA, Hussein OA, Ahmed W, Abdelrazek AH, Homod RZ (2022). Thermohydraulic analysis of covalent and noncovalent functionalized graphene nanoplatelets in circular tube fitted with turbulators. Sci. Rep..

[24] Wang A, Long L, Zhao W, Song Y, Humphrey MG, Cifuentes MP (2013). Increased optical nonlinearities of graphene nanohybrids covalently functionalized by axially-coordinated porphyrins. Carbon.

[25] Zhao C, He C, Dong Y, Song W (2019). The third order nonlinear optical properties of graphene oxide–zinc (II) naphthalocyanine hybrids and amino graphene oxide–zinc (II) naphthalocyanine hybrids. Carbon.

[26] Zhao H, Yang J, Wang L, Tian C, Jiang B, Fu H (2011). Fabrication of a palladium nanoparticle/graphene nanosheet hybrid via sacrifice of a copper template and its application in catalytic oxidation of formic acid. Chem. Commun..

[27] Ramaraju B, Li C-H, Prakash S, Chen C-C (2016). Metal–organic framework derived hollow polyhedron metal oxide posited graphene oxide for energy storage applications. Chem. Commun..

[28] Anasori B, Beidaghi M, Gogotsi Y (2014). Graphene–transition metal oxide hybrid materials. Mater. Today.

[29] Wu Z, Zhou GM, Yin L-C, Ren WC, Li F, Cheng H-M (2012). Graphene/metal oxide composite electrode materials for energy storage. Nano Energy.

[30] Fang X, Liu J, Wang J, Zhao H, Ren H, Li Z (2017). Dual signal amplification strategy of Au nanopaticles/ZnO nanorods hybridized reduced graphene nanosheet and multienzyme functionalized Au@ ZnO composites for ultrasensitive electrochemical detection of tumor biomarker. Biosens. Bioelectron..

[31] Lee S, Oh J, Kim D, Piao Y (2016). A sensitive electrochemical sensor using an iron oxide/graphene composite for the simultaneous detection of heavy metal ions. Talanta.

[32] Anand K, Singh O, Singh MP, Kaur J, Singh RC (2014). Hydrogen sensor based on graphene/ZnO nanocomposite. Sens. Actuators B Chem..

[33] Xiong S, Ye S, Hu X, Xie F (2016). Electrochemical detection of ultra-trace Cu (II) and interaction mechanism analysis between amine-groups functionalized CoFe2O4/reduced graphene oxide composites and metal ion. Electrochim. Acta.

[34] Xu C, Wang X, Zhu J, Yang X, Lu L (2008). Deposition of Co3 O4 nanoparticles onto exfoliated graphite oxide sheets. J. Mater. Chem..

[35] Hung LI, Tsung CK, Huang W, Yang P (2010). Room-temperature formation of hollow Cu2O nanoparticles. Adv. Mater..

[36] Zou W, Zhu J, Sun Y, Wang X (2011). Depositing ZnO nanoparticles onto graphene in a polyol system. Mater. Chem. Phys..

[37] Wang X, Wu H-F, Kuang Q, Huang R-B, Xie Z-X, Zheng L-S (2010). Shape-dependent antibacterial activities of Ag2O polyhedral particles. Langmuir.

[38] Wu X-L, Wang L, Chen C-L, Xu A-W, Wang X-K (2011). Water-dispersible magnetite-graphene-LDH composites for efficient arsenate removal. J. Mater. Chem..

[39] Ghorashi MS, Madaah Hosseini HR, Mohajerani E, Pedroni M, Taheri Ghahrizjani R (2021). Enhanced TiO2 broadband photocatalytic activity based on very small upconversion nanosystems. J. Phys. Chem. C.

[40] Ghahrizjani RT, Yousefi MH (2017). Effects of three seeding methods on optimization of temperature, concentration and reaction time on optical properties during growth ZnO nanorods. Superlattices Microstruct..

[41] Sharifi Malvajerdi S, Abrari M, Karimi V, Shafiee M, Ghollamhosseini S, Taheri Ghahrizjani R (2021). High-voltage, high-current electrical switching discharge synthesis of ZnO nanorods: A new method toward rapid and highly tunable synthesis of oxide semiconductors in open air and water for optoelectronic applications. ACS Appl. Mater. Interfaces.

[42] Chen M-T, Lu M-P, Wu Y-J, Song J, Lee C-Y, Lu M-Y (2010). Near UV LEDs made with in situ doped pn homojunction ZnO nanowire arrays. Nano Lett..

[43] McCune M, Zhang W, Deng Y (2012). High efficiency dye-sensitized solar cells based on three-dimensional multilayered ZnO nanowire arrays with “caterpillar-like” structure. Nano Lett..

[44] Zhou W, Zhang J, Liu Y, Li X, Niu X, Song Z (2008). Characterization of anti-adhesive self-assembled monolayer for nanoimprint lithography. Appl. Surf. Sci..

[45] Ameri M, Ghaffarkani M, Ghahrizjani RT, Safari N, Mohajerani E (2020). Phenomenological morphology design of hybrid organic–norganic perovskite solar cell for high efficiency and less hysteresis. Sol. Energy Mater. Sol. Cells.

[46] Azadinia M, Ameri M, Ghahrizjani RT, Fathollahi M (2021). Maximizing the performance of single and multijunction MA and lead-free perovskite solar cell. Mater. Today Energy.

[47] Ghahrizjani RT, Ghafarkani M, Janghorban S, Ameri M, Azadinia M, Mohajerani E (2021). ZnO–SrAl2O4: Eu nanocomposite-based optical sensors for luminescence thermometry. ACS Appl. Nano Mater..

[48] Amirsalari A, Ziabari AA, Ghahrizjani RT, Shayesteh SF (2017). A fundamental study on the effects of nano-silver incorporation on the structure and luminescence properties of color centers in γ′-alumina nanoparticles. J. Lumin..

[49] Sadrolhosseini AR, Ghasami E, Pirkarimi A, Hamidi SM, Ghahrizjani RT (2022). Highly sensitive surface plasmon resonance sensor for detection of methylene blue and methylene orange dyes using NiCo-layered double hydroxide. Opt. Commun..

[50] Hu Y, Zhou J, Yeh PH, Li Z, Wei TY, Wang ZL (2010). Supersensitive, fast-response nanowire sensors by using Schottky contacts. Adv. Mater..

[51] Wang K, Li M, Zhang J, Lu H (2019). Polyacrylonitrile coupled graphite oxide film with improved heat dissipation ability. Carbon.

[52] Gao W (2015). The Chemistry of Graphene Oxide.

[53] Hofmann U, Holst R (1939). Über die Säurenatur und die Methylierung von Graphitoxyd. Berichte der deutschen Chem. Gesellschaft (A and B Ser.).

[54] Jaleh B, Jabbari A (2014). Evaluation of reduced graphene oxide/ZnO effect on properties of PVDF nanocomposite films. Appl. Surf. Sci..

[55] Marcano DC (2010). Improved synthesis of graphene oxide. ACS Nano.

[56] Jasim DA, Lozano N, Kostarelos K (2016). Synthesis of few-layered, high-purity graphene oxide sheets from different graphite sources for biology. 2D Mater..

[57] McAllister MJ, Li J-L, Adamson DH, Schniepp HC, Abdala AA, Liu J (2007). Single sheet functionalized graphene by oxidation and thermal expansion of graphite. Chem. Mater..

[58] Luo Z, Lu Y, Somers LA (2009). ATC Johnson-high yield preparation of macroscopic graphene oxide membranes. J. Am. Chem. Soc..

[59] Khanra P, Kuila T, Kim N, Bae S, Sheng YD, Lee JH (2012). Simultaneous bio-functionalization and reduction of graphene oxide by baker’s yeast. Chem. Eng. J..

[60] Wang M, Tan G, Ren H, Xia A, Liu Y (2019). Direct double Z-scheme Og-C3N4/Zn2SnO4N/ZnO ternary heterojunction photocatalyst with enhanced visible photocatalytic activity. Appl. Surf. Sci..

[61] Kuila T, Bose S, Khanra P, Mishra AK, Kim NH, Lee JH (2011). Recent advances in graphene-based biosensors. Biosens. Bioelectron..

[62] Eda G, Chhowalla M (2010). Chemically derived graphene oxide: Towards large-area thin-film electronics and optoelectronics. Adv. Mater..

[63] Prabhu S, Megala S, Harish S, Navaneethan M, Maadeswaran P, Sohila S (2019). Enhanced photocatalytic activities of ZnO dumbbell/reduced graphene oxide nanocomposites for degradation of organic pollutants via efficient charge separation pathway. Appl. Surf. Sci..

[64] Rodwihok C, Choopun S, Ruankham P, Gardchareon A, Phadungdhitidhada S, Wongratanaphisan D (2019). UV sensing properties of ZnO nanowires/nanorods. Appl. Surf. Sci..

[65] Kubo R (1962). Electronic properties of metallic fine particles. I. J. Phys. Soc. Jpn..

[66] Perumbilavil S, Sridharan K, Koushik D, Sankar P, Pillai VM, Philip R (2017). Ultrafast and short pulse optical nonlinearity in isolated, sparingly sulfonated water soluble graphene. Carbon.

[67] Song W, He C, Zhang W, Gao Y, Yang Y, Wu Y (2014). Synthesis and nonlinear optical properties of reduced graphene oxide hybrid material covalently functionalized with zinc phthalocyanine. Carbon.

[68] Li P-L, Wang Y-H, Shang M, Wu L-F, Yu X-X (2020). Enhanced optical limiting properties of graphene oxide-ZnS nanoparticles composites. Carbon.

[69] Majles Ara M, Dehghani Z (2012). Measurement of nonlinear responses and optical limiting behavior of Tio2/Ps nano-composite by single beam technique with different incident intensities. Int. J. Mod. Phys. Conf. Ser..

[70] Singh V, Aghamkar P, Lal B (2013). Third-order nonlinear optical properties and reverse saturable absorption in 2, 3-butanedione dihydrazone using z-scan technique. Acta Phys. Polon. A.

